# AI-Based Prediction and Prevention of Psychological and Behavioral Changes in Ex-COVID-19 Patients

**DOI:** 10.3389/fpsyg.2021.782866

**Published:** 2021-12-28

**Authors:** Krešimir Ćosić, Siniša Popović, Marko Šarlija, Ivan Kesedžić, Mate Gambiraža, Branimir Dropuljić, Igor Mijić, Neven Henigsberg, Tanja Jovanovic

**Affiliations:** ^1^Faculty of Electrical Engineering and Computing, University of Zagreb, Zagreb, Croatia; ^2^Croatian Institute for Brain Research, University of Zagreb School of Medicine, Zagreb, Croatia; ^3^Department of Psychiatry and Behavioral Neurosciences, Wayne State University School of Medicine, Detroit, MI, United States

**Keywords:** artificial intelligence, mental health disorders, prediction and prevention, ex-COVID-19 patients, semantic/acoustic features, neurophysiological features, facial/oculometric features

## Abstract

The COVID-19 pandemic has adverse consequences on human psychology and behavior long after initial recovery from the virus. These COVID-19 health sequelae, if undetected and left untreated, may lead to more enduring mental health problems, and put vulnerable individuals at risk of developing more serious psychopathologies. Therefore, an early distinction of such vulnerable individuals from those who are more resilient is important to undertake timely preventive interventions. The main aim of this article is to present a comprehensive multimodal conceptual approach for addressing these potential psychological and behavioral mental health changes using state-of-the-art tools and means of artificial intelligence (AI). Mental health COVID-19 recovery programs at post-COVID clinics based on AI prediction and prevention strategies may significantly improve the global mental health of ex-COVID-19 patients. Most COVID-19 recovery programs currently involve specialists such as pulmonologists, cardiologists, and neurologists, but there is a lack of psychiatrist care. The focus of this article is on new tools which can enhance the current limited psychiatrist resources and capabilities in coping with the upcoming challenges related to widespread mental health disorders. Patients affected by COVID-19 are more vulnerable to psychological and behavioral changes than non-COVID populations and therefore they deserve careful clinical psychological screening in post-COVID clinics. However, despite significant advances in research, the pace of progress in prevention of psychiatric disorders in these patients is still insufficient. Current approaches for the diagnosis of psychiatric disorders largely rely on clinical rating scales, as well as self-rating questionnaires that are inadequate for comprehensive assessment of ex-COVID-19 patients’ susceptibility to mental health deterioration. These limitations can presumably be overcome by applying state-of-the-art AI-based tools in diagnosis, prevention, and treatment of psychiatric disorders in acute phase of disease to prevent more chronic psychiatric consequences.

## Introduction

The COVID-19 pandemic has created enormous stressors across the globe by elevating rates of anxiety, depression, posttraumatic stress disorder (PTSD), or even suicidal behaviors (Bäuerle et al., 2020; Sherman et al., 2020; Wu T. et al., 2020; Cheung et al., 2021; Czeisler et al., 2021). The inadequate number of psychiatrists, lack of predictive biomarkers (Walker et al., 2017), and human subjectivity in assessments can further aggravate these problems, leading to a surge in the incidence and prevalence of mental health disorders across the globe. Therefore, urgent actions on extensive monitoring of mental health are needed, particularly concerning prediction and early treatment of individuals who were exposed to a high level of COVID-19-related distress, especially if they exhibit low overall stress resilience (Riehm et al., 2021) and other specific vulnerabilities (Li et al., 2020; Tuman, 2021). Recognition and identification of such high-risk individuals in the early stages of acute stress is extremely important to prevent the development of more serious long-term mental health disorders. The proposed predictive methodology presented in this article is based on appropriate multimodal stimuli, corresponding multimodal neuro-psycho-physiological features and their AI-based integration and analysis. A new generation of AI-based tools emerging in psychiatry (Ćosić et al., 2020a,b) might facilitate prediction of risk of chronic psychopathology (Karstoft et al., 2015; Schultebraucks et al., 2020a) and detection of suicidal ideation (Just et al., 2017), enable automated delivery of treatment (Fitzpatrick et al., 2017), help understand crucial ingredients of psychotherapy (Ewbank et al., 2020), as well as destigmatize reporting of mental health symptoms as virtual human interviewers (Lucas et al., 2017). Therefore, whether in prediction, prevention, diagnosis or treatment, AI-based technologies exhibit transformational potential to shift a self-report, conversation-, and observation-based qualitative system in psychiatry toward a more data/information-rich and quantitative one, even if several challenges still exist along this path (Graham et al., 2019; Rainey and Erden, 2020). A major strength of statistical and AI methods is their ability to identify specific non-obvious patterns beyond human computational capabilities within highly heterogeneous multimodal sets of data relevant for mental health assessment (Eyre et al., 2016; Garcia-Ceja et al., 2018), which is essential for early detection of ex-COVID-19 patients at high risk of mental health deterioration. Diverse forms of data for inclusion into such heterogeneous data sets have been encountered in several publications on AI in mental health (Eyre et al., 2016; Garcia-Ceja et al., 2018; Graham et al., 2019): various rating scales used by patients and clinicians, electronic health records (EHRs), brain imaging data, genomics, blood biomarkers, data collected while using smartphones, textual posts from social media platforms, speech and language audio data, facial video data, multiple peripheral physiological signals etc. Assuming that mentally vulnerable individuals affected by COVID-19 pandemic vs. resilient individuals have different profile of neuro-psycho-physiological indicators together with differences in personality traits and psycho-behavioral status, a comprehensive AI-based predictive approach should lead to better detection of high-risk ex-COVID-19 patients (Ramos-Lima et al., 2020) and more effective prevention strategies. The following sections present the concept of prediction of potential chronic mental health diseases among ex-COVID-19 patients, which includes comprehensive psychological, neurophysiological, semantic, acoustic, and facial/oculometric measurements and features, as well as the concept of mental health disorder prevention using AI methods.

## Prediction

The main objective of the proposed methodology for prediction of potential mental health disorders among ex-COVID-19 patients is to extend clinical interview and self-report information with more objective metrics. Therefore, this section provides an overview of a variety of psychological, speech-based, neurophysiological, as well as facial/oculometric features as potential predictors of mental health deterioration in the setting of post-COVID clinics. Such comprehensive data obtained through extended screening procedures of ex-COVID-19 patients in post-COVID clinics (Rubin, 2020; Walter, 2021), analyzed with AI-based tools of unsupervised machine learning methods (such as multivariate correlation analyses, clustering, and principal component analysis), as well as a range of traditional supervised machine learning methods (such as artificial neural networks, support vector machines and contemporary deep learning supervised approaches) can significantly improve prediction accuracy. A variety of AI-based tools which are already being used in research on prediction of adverse mental health outcomes from diverse data sets, are covered in the subsection on “Multimodal data fusion: statistical and ML analysis.”

### Psychological Features

Understanding that self-report psychological screening is also important, we will briefly discuss these instruments for prediction of chronic psychopathology. A wide variety of self-report questionnaires, many of which have already been employed in COVID-19-related mental health research, is potentially applicable within the proposed prediction methodology:

• Questionnaires regarding overall mental health and wellbeing, like the General Health Questionnaire (GHQ-12) (Gao et al., 2004; Pierce et al., 2020; Veer et al., 2021) and the Short Form Health Survey questionnaires (SF-36 and SF-12) (Ware and Sherbourne, 1992; Ware et al., 1996; Chen et al., 2020; Zhang S. X. et al., 2020).
• Questionnaires regarding stress resilience as an important protective factor against chronic psychopathology, e.g., the Connor-Davidson Resilience Scale (Connor and Davidson, 2003; Cai et al., 2020) and the Brief Resilience Scale (Smith et al., 2008; Riehm et al., 2021), as well as questionnaires targeting other related protective or risk factors, like hardiness (Eschleman et al., 2010; Rezaei and Vakilian, 2021), distress tolerance (Simons and Gaher, 2005; Liu N. et al., 2020), appraisal style (Kalisch et al., 2015; Veer et al., 2021), self-control (Maloney et al., 2012; Li et al., 2020), trait anxiety (Speilberger and Vagg, 1984; Lin et al., 2020), anxiety sensitivity (Deacon et al., 2003; Warren et al., 2021), and type D personality (Denollet, 2005; Tuman, 2021).
• Questionnaires regarding preexisting vulnerability to psychopathology that stems from adverse experiences encountered in life prior to pandemic, such as Life Events Checklist (Gray et al., 2004; Marco et al., 2020), Adverse Childhood Experiences Questionnaire (ACE-Q) (Felitti et al., 1998; Zarse et al., 2019; McCarthy et al., 2021), and the Trauma History Questionnaire (Hooper et al., 2011; Kolacz et al., 2020).
• Questionnaires closely aligned with diagnostic criteria for specific mental health disorders, like PTSD Checklist for DSM-5 (PCL-5) (Blevins et al., 2015; Liu N. et al., 2020), Impact of Event Scale–Revised (Weiss, 2004; Wang et al., 2020), Beck Depression Inventory (Beck et al., 1961; Di Tella et al., 2020), Generalized Anxiety Disorder scale (GAD-7) (Spitzer et al., 2006; Bäuerle et al., 2020). These questionnaires are an essential part of any future predictive algorithm, since a very high score on any of them suggests an immediate risk that the disorder is already emerging or present.
• Questionnaires regarding general distress experienced by ex-COVID-19 patients during the pandemic, like K10 Kessler Psychological Distress Scale (Andrews and Slade, 2001; Janiri et al., 2020), Perceived Stress Scale (Roberti et al., 2006; Eubank et al., 2021), and Peritraumatic Dissociative Experiences Questionnaire (Marmar et al., 1997; Ranieri et al., 2021).
• Questionnaires on COVID-19-specific distress, which are either adapted from existing illness-related questionnaires, like Brief Illness Perception Questionnaire (Broadbent et al., 2006; Hubbard et al., 2021) or newly developed, e.g., Fear of COVID-19 Scale (Ahorsu et al., 2020; Bitan et al., 2020), Post-COVID-19 Functional Status Scale (Klok et al., 2020), and COVID-19 Peritraumatic Distress Index (CPDI) (Qiu et al., 2020).

Finally, self-reports regarding the presence of a previously diagnosed psychopathology (Asmundson et al., 2020) would be important in cases where mental health history is not otherwise available, since it is known that depression and anxiety occur more commonly in patients with previous psychiatric diagnosis (Hao et al., 2020). In selection of questionnaires in line with this brief review, it is important to minimize their overlap, while favoring all of those with strong scientific evidence as risk assessment instruments, to acquire efficiently the most valuable psychological features for prediction of future psychopathology.

### Speech Features

The potential of human speech patterns in detection and prediction of psychopathology has already been demonstrated in several research works (Cummins et al., 2015; Marmar et al., 2019; Schultebraucks et al., 2020b). Therefore, this section discusses speech features that are the most promising for application in risk assessment of ex-COVID-19 patients. A wide range of methods to elicit speech features for assessment and prediction of mental health disorders include: vocal exercises; reading neutral or emotionally charged words, sentences or passages, with or without mood induction by pictures or videos; as well as free-form speaking while describing emotionally arousing events or during interviews conducted by humans or virtual agents (Cummins et al., 2015). Due to prior experiences that free-form speech might offer better prediction performance compared to a read passage (Cummins et al., 2015), free-form speech elicitation methods might be preferable for ex-COVID-19 patients. While free-form speech collection during time-consuming disorder-specific diagnostic interviews can provide valuable speech information, e.g., in PTSD assessment (Marmar et al., 2019), it would require excessive time to conduct multiple diagnostic interviews for a broader range of COVID-19-relevant mental health problems, like generalized anxiety, depression, PTSD and suicidal ideation/behavior. Accordingly, for elicitation of speech in ex-COVID-19 patients, it might be more appropriate to use: (a) relevant modules of shorter structured clinical interviews, like M.I.N.I. (Sheehan et al., 1998; Winkler et al., 2020); (b) semi-structured interviews on COVID-19 experiences, which have been applied in qualitative research during the pandemic (Gillard et al., 2021; McKinlay et al., 2021); (c) COVID-19-related adaptations of very brief time-limited interviews, like the 3-min interview from Schultebraucks et al. (2020b); or (d) COVID-19-related concretizations of broad interview formats that are generally structured around 5W and H questions, e.g., (Dilts, 1990).

#### Semantic Features

More accurate prediction of potential psychiatric disorders among ex-COVID-19 patients would benefit from AI-based natural language processing (NLP) of free-form speech to make distinctions between stress vulnerable vs. stress resilient ex-COVID-19 patients. Such linguistic information and related unstructured data might be of great importance as valuable clinical information to identify relevant keywords for early detection of various mental health problems. NLP can capture patterns of irrational or distorted language associated with patterns of specific psychopathologies, like patient’s mental distortions, biases, core beliefs, negative wording, and compensatory strategies. Using NLP techniques, we can uncover patterns of how specific psychopathologies are reflected in language, and related semantic and acoustic features across time, what might be helpful in post-COVID clinical settings. From a technical perspective, all answers, comments and observations during the interview are continuously monitored and serve as a basis for real-time, and subsequently off-line, NLP analysis. To support this, an automated conversion of microphone-recorded speech to text is performed in the background.

Many studies analyze speech content in terms of linguistic measures such as word frequencies, lexical diversity, narrative coherence, sentiment of speech content and others, using them as features to classify major depressive disorder (MDD) (Cummins et al., 2011, 2015; Alghowinem et al., 2013; Calvo et al., 2017; Schultebraucks et al., 2020b) and PTSD (Scherer et al., 2015; He et al., 2017; Schultebraucks et al., 2020b). Schultebraucks et al. (2020b) showed that the most important features for predicting PTSD and MDD status were the NLP features, while the features from other groups (face, acoustic and movement) also showed high potential. Features were calculated using the Linguistic Inquiry and Word Count (LIWC) 2015 dictionary (Pennebaker et al., 2015) and DeepSpeech (Hannun et al., 2014) from free-form speech responses to a brief interview 1 month after the trauma. Predictive importance ranking was done using SHAP (SHapley Additive exPlanation). The most important predictor for PTSD was NLP LIWC “self-assured,” while for MDD, it was age, followed by NLP LIWC “workhorse.”

LIWC is a widely used software tool in mental health projects (Calvo et al., 2017). It is composed of more than 6,000 words, word stems and selected emoticons and calculates approximately 90 output variables for each text input, i.e., word count, summary language variables (e.g., authenticity, emotional tone), general descriptor categories (e.g., words per sentence), word categories tapping psychological constructs (e.g., affect, cognition, biological processes), informal language markers (e.g., assents, fillers, swear words, netspeak) and others (Pennebaker et al., 2015). In addition to LIWC, authors often use other linguistic tools such as N-gram language models, emotional thesauri like WordNet-affect (Strapparava and Valitutti, 2004) and normative databases such as the Affective Norm for English Words (ANEW) (Bradley and Lang, 1999).

A study was done on the topic of wording trend analysis and other NLP analyzes in 15 of the world’s largest mental health support groups found on Reddit (e.g., r/schizophrenia, r/SuicideWatch, r/Depression) during the initial stage of the COVID-19 pandemic (Low et al., 2020). Beside the LIWC features, the authors also included sentiment metrics, basic word and syllable counts, punctuation, readability metrics, term frequency–inverse document frequency (TF-IDF) ngrams and manually built lexicons about suicidality, economic stress, isolation, substance use, domestic stress and guns. Although the paper is not directly related to the prediction or diagnosis of mental health disorders in ex-COVID-19 patients, the presented textual features and conclusions of the analyses can be used as valuable information for tuning and improving such prediction and diagnosis models.

Most recent studies in the field of mental health-related NLP, as shown above, are still based on the analysis of keywords and statistical representations of words, although NLP has been elevated to the context-based semantic level of word representations in the last few years. The reason behind this is mainly due to the ease of interpretation of keyword-based features, while on the other hand, the context-based representation of words is more abstract.

In general, this semantic NLP progress can be shown through the following most important achievements: *bag-of-words* (word-count metrics that ignore grammatical structure) (Harris, 1954), *word2vec* (word embeddings—static representation of words in a semantic multidimensional space based on the context in which these words occur in a specific language) (Mikolov et al., 2013), and *BERT* (transformer-based language model that represents words and subwords through contextual embeddings in a way that they dynamically change their position in the semantic space depending on the specific environment, e.g., sentence, in which they are located) (Devlin et al., 2019).

#### Acoustic Features

Acoustic features of speech have widely been used as indirect markers of underlying physiological phenomena. These features are useful within the clinical interview paradigm, described at the beginning of this section, as speech production involves both simultaneous cognitive planning and complex motoric muscular actions (Cummins et al., 2015). Slight physiological and cognitive changes can produce noticeable acoustic changes (Scherer, 1986) in the perceived speech during such events. Examples of such changes were documented as far as 1921 (Kraepelin, 1921), where voices of depressed patients were described as low, slow, hesitant and monotonous (amongst other things). While some of those descriptions can easily be explained with singular features, e.g., lower speech fundamental frequency (sometimes called pitch) in the case of low voice, or lower number of voiced frames in an utterance (speech rate) and pauses between words in the case of slow voice, we often find more complex groups of features describing such phenomena as they can produce more information for such a varied process like speech. Such features can also help to identify other breathing abnormalities (e.g., Mukherjee et al., 2021a,b).

In our previous research we have focused on developing optimized stimulation paradigms for elicitation of multimodal responses related to stress resilience (Dropuljić et al., 2017; Ćosić et al., 2019b) and cognitive functioning estimation (Mijić et al., 2017, 2019). Acoustic feature sets were related to: (a) fundamental frequency, an estimate of the base harmonic that the vibrating vocal cords are producing during vocalization; RMS energy, a signal-processing-based estimate of the energy of the sound recorded by the microphone; (b) formant frequencies (f1–f4) and mel-frequency-cepstral-coefficients which describe the spectral and cepstral behavior of the recorded utterances; (c) jitter and shimmer, representing voice perturbations; and (d) number of voiced segments per second and mean voiced/unvoiced segment lengths in seconds, which are related to speech rate.

Predefined experimental paradigms (e.g., Dropuljić et al., 2017; Ćosić et al., 2019b) may be applicable for collecting speech features that would be helpful in identifying ex-COVID-19 patients suffering from post-COVID-19-related psychopathology. However, we believe that using utterances from clinical interviews would be more beneficial as it enables a more “close-nit” integration with semantic features and free-form speaking has evident advantages when collecting speech data in the context of psychopathologies (Cummins et al., 2015). Afshan et al. (2018) describe a particularly similar usecase, where depressed vs. normal speech was successfully classified using speech recordings of 1688 clinical interviews (735 depressive and anxiety disorders, 953 healthy). The used feature sets greatly overlapped with the ones we used in our stress resilience and cognitive load estimation research, described in the previous paragraph. A comprehensive framework for analyzing depression in the context of voice features is presented in Cummins et al. (2015), alongside an exhaustive overview of papers that deal with particular voice features and how they relate to either the physiological or cognitive effects of depressive disorders. Such body of work provides the necessary interpretability needed in usecases where we deploy the above-described features as diagnostic tools for usecases presented in this paper. An important property of speech as a modality is that it can easily and reliably be collected within the paradigm proposed in this paper, and can serve within the multimodal framework, complementing other modalities.

In summary, speech as a modality within the clinical interview paradigm, and as a component of a larger set of paradigms would prove a suitable augmentation alongside semantic, neurophysiological, and facial/oculometric features. Acoustic speech analysis would be particularly valuable alongside semantic analysis of the clinical interview, for prediction of potential mental health disorders like depression and early identification of various mental health risks in ex-COVID-19 patients. Furthermore, acoustic speech analysis may potentially be used as a real-time analysis tool that provides valuable information about the dynamics of the patients’ emotional change during AI-based therapy described in section on Prevention, or a tool that helps show differences between resilient vs. vulnerable patients.

### Neurophysiological Features

This section provides an overview of multimodal neurophysiological features as potential predictors of mental health deterioration in ex-COVID-19 patients. The main idea is a comprehensive set of carefully selected, elicited, and quantified neurophysiological features which can help identify those ex-COVID-19 patients who are at risk of developing post-COVID-19-related psychopathology, such as depression, anxiety, or PTSD (Liu C. H. et al., 2020; Mazza et al., 2020). These features are assessed during specific test paradigms and tasks which elicit corresponding multimodal responses and can be associated with patients’ neuro-psycho-physiological risk states, like acute stress, anxiety (Shaffer and Ginsberg, 2017; Arza et al., 2019), and cognitive decline (Lynch and Lachman, 2020); as well as protective traits, like stress resilience (Walker et al., 2017). Translation of earlier findings, particularly in the area of physiology-based resilience/vulnerability assessment (Ćosić et al., 2019a; Šarlija et al., 2021), as well as in the area of objective neurophysiological assessment of cognitive functioning (Kesedžić et al., 2020a), could facilitate the design of efficient and reliable methods for multimodal prediction of potentially vulnerable individuals among ex-COVID-19 patients.

Stress resilience can be defined as an ability or a process of maintaining normal psychological, physiological, and physical functioning when exposed to high levels of stress and trauma (Russo et al., 2012). Moreover, a review of various objective biomarkers of stress resilience by Walker et al. (2017), described resilience as the absence of trauma-related psychiatric disorder symptoms, i.e., PTSD, as well as of other disorders like depression (Edward, 2005; Schultebraucks et al., 2021). A primary vision highlighted in the review by Walker et al. (2017), as well by our research on multimodal physiology-based stress resilience assessment (Ćosić et al., 2019a; Šarlija et al., 2021), is the “early identification of individuals who are at risk of developing PTSD or depression” (Walker et al., 2017), which becomes particularly valuable in the face of the COVID-19 pandemic (Ćosić et al., 2020a; Liu C. H. et al., 2020; Mazza et al., 2020).

The deficits in a wide range of cognitive processes and/or dysfunction of the prefrontal cortex (PFC) and its connected circuitry are already associated with many mental disorders and neurological conditions (Friedman and Robbins, 2021). The cognitive decline in ex-COVID-19 patients, related to cognitive deficits (Hampshire et al., 2021), as well as global cognitive impairment, impairment in memory, attention, and executive function (Daroische et al., 2021), is one of the specific characteristics of mental health vulnerabilities among the ex-COVID-19 patients. Cognitive assessment of patients with recent COVID-19 infection through specialized clinics dedicated to further diagnostic assessment and tailored rehabilitation is recognized as a preventive measure of mental health deterioration (Liotta et al., 2020; Daroische et al., 2021).

#### Peripheral Physiological Features

Peripheral physiological features of interest are mainly related to resting autonomic nervous system (ANS) activity, like resting heart rate variability (HRV) and respiratory sinus arrhythmia (RSA); features of psychophysiological allostasis, like cardiac allostasis; as well as electromyogram- and electrodermal activity-based (EMG and EDA) acoustic startle response (ASR) features, like startle reactivity, startle habituation, and startle-modulation-related features, e.g., fear/anxiety-potentiated or prepulse inhibited startle response (Ćosić et al., 2019a; Šarlija et al., 2021). In the following paragraphs, we aim to expand on our prior research related to identification and assessment of various multimodal physiological features of stress resilience (Ćosić et al., 2019a; Šarlija et al., 2021) in the direction of potential discriminators of ex-COVID-19 patients which are either resilient or at-risk for the development of COVID-19-related psychopathology.

In our research on objectivization of stress resilience assessment (Ćosić et al., 2019a), several physiological features confirmed discriminative power between resilient and non-resilient mentally healthy individuals. These were related to: (a) resting RSA, which measures HRV in phase with inhalation and exhalation (Grossman and Taylor, 2007); (b) EMG-based startle reactivity, which measures the strength of reflexive defensive responding to an aversive unconditioned stimulus, i.e., abrupt, loud noise (Bradford et al., 2014); and (c) cardiac allostasis, which measures adaptive reaction to a stressful event, involving a vigorous cardiac response to stress coupled with a significant cardiac recovery in the aftermath (Souza et al., 2013). These three simple physiological features were also recognized as potential biomarkers of stress resilience according to an extensive review of relevant prior research on the psychophysiology of resilience (Walker et al., 2017).

This paragraph provides a brief overview of physiological features supported by evidence from prospective studies, which are in the context of the current paper considered the most relevant category of literature. A simple EDA-based measure of sympathetic activity, collected during a standard trauma interview in the immediate aftermath of a traumatic event, was shown to predict risk of developing PTSD (Hinrichs et al., 2019). Elevated heart rate following a traumatic event is a simple physiological feature that was associated with later development of warzone-related PTSD (Shalev et al., 1998; Dean et al., 2019). Both increased heart rate and reduced high frequency HRV (HF-HRV) predicted PTSD development following a trauma exposure in women (Seligowski et al., 2021). Another simple measure of resting vagally-mediated HRV, i.e., RMSSD, was negatively associated with development of depressive symptoms in healthy young subjects, over a period of 3 years (Carnevali et al., 2018). HF-HRV and RMSSD are, besides RSA, also features which are linked to resting autonomic nervous system activity. Startle reactivity, introduced above as a marker of resilience, as well as other ASR-related features, e.g., startle habituation, were also found to be predictive of PTSD development (Shalev et al., 2000; Pole et al., 2009; Risbrough et al., 2012, 2017). In the context of COVID-19, EMG-based startle reactivity, interpreted as a physiological marker of anxiety was increased in COVID-19 physicians compared to non-COVID-19 physicians, and was also predictive of later anxiety scores measured by GAD-7 (Dolev et al., 2021). A systematic literature review on the association between HRV reactivity to laboratory stress (which is similar to the concept of cardiac allostasis, mentioned in the paragraph above) and depression, states that none of reviewed studies have evaluated the prospective prediction of depressive symptoms (Hamilton and Alloy, 2016). The authors claim that this “will be important in differentiating whether HRV reactivity is a consequence or correlate of depression or a marker of vulnerability to future depression,” which is in line with our discussion on the importance of prospective research. Besides physiological features, other markers such as polygenic scores (Schultebraucks et al., 2021), cortisol (Galatzer-Levy et al., 2017), or measures of hippocampal activity (van Rooij et al., 2018, 2021) were also identified as predictive of mental health deterioration after major life stressors, but a review of such an expanded set of potential predictors exceeds the scope of this section. A vast amount of research was also done on various other physiological features as correlates or diagnostic markers of PTSD, depression, anxiety, etc. but are here omitted due to their already mentioned decreased relevance in comparison with prospective research.

A brief literature review presented above demonstrates the potential of simple yet informative physiological features like resting RSA or EMG-based startle reactivity, which can be elicited and assessed using a relatively short and generic stimulation and evaluation protocol (Ćosić et al., 2019a,b; Šarlija et al., 2021). Therefore, the proposed protocol for elicitation and assessment of physiological features in the prediction of mental health deterioration in ex-COVID-19 patients should include a resting period, a stress-inducing period with acoustic startle stimuli, and a post-stress recovery period, while the patients’ peripheral physiological responses are being continuously recorded.

#### Prefrontal Cortex Physiological Features

Cognitive decline, which is observable in the PFC activation on a variety of cognitive tasks, is one of the characteristics of mental health vulnerabilities in ex-COVID-19 patients (Daroische et al., 2021; Hampshire et al., 2021). The prediction of vulnerable individuals among the ex-COVID-19 patients can be improved using brain monitoring techniques, and particularly features extracted from the PFC activation. The neuroanatomical connectivity of the PFC to most parts of the cortical and subcortical brain makes it well suited for participating in a number of neural networks and carrying out cognitive control operations in different functional domains (e.g., spatial, visual, and verbal) (Friedman and Robbins, 2021). The PFC is linked through its extensive association connections with distant and broadly dispersed parts of the association and limbic cortices and these interconnections with the amygdala, hypothalamus, midbrain, and pons represent important subcortical linkages of the extended prefrontal neural system.

Research on mental disorders using brain imaging techniques, including functional near-infrared spectroscopy (fNIRS), a simple, wearable, and low-cost brain imaging technique which enables PFC activation estimation, suggests that these disorders change the way the brain functions during different cognitive challenges. fNIRS was used to estimate the PFC activation on a variety of cognitive tasks in patients with PTSD (Matsuo et al., 2003; Tian et al., 2014; Yennu et al., 2016; Gramlich et al., 2017), panic disorder (Nishimura et al., 2007), social anxiety disorder (Yokoyama et al., 2015), and MDD (Nishizawa et al., 2019; Zhu et al., 2020). Differences between these groups of patients and healthy individuals were found, suggesting a hypoactivation of the PFC in individuals with PTSD compared to healthy controls in verbal fluency task (Matsuo et al., 2003), as well as hypoactivation in the left lateral PFC during the incongruent Stroop task (Yennu et al., 2016). A general conclusion from these studies is that PTSD patients experience PFC hypoactivation during cognitive tasks, but their PFC activation increases dramatically when the person encounters an object associated with tragic and traumatic events (Kim et al., 2017). A systematic review by Ho et al. (2020) on applications of fNIRS for MDD indicated that fNIRS consistently demonstrated attenuated cerebral hemodynamic changes in depressed compared to healthy individuals. Patients with MDD or panic disorder show hypoactivation in the PFC during cognitive tasks (Kim et al., 2017), while high-trait-anxiety individuals showed a reduced PFC activation during the cognitive task compared to those with low anxiety (Brugnera et al., 2017). In our research on cognitive functioning, we classified different cognitive states using neurophysiological signals (Kesedžić et al., 2020a), and showed differences in brain activation among individuals during cognitive tasks and discussed the applicability of such objective monitoring systems in a real-working environment (Kesedžić et al., 2020b). In addition to the research in patients with mental disorders and healthy controls, the fNIRS was used on ex-COVID-19 patients in the research of hypoxemia (Ferrari and Quaresima, 2020) as well as during presentation of olfactory stimuli (Ho et al., 2021).

In summary, the tasks used in research include verbal fluency tasks, Stroop tasks, mathematical tasks, neutral and fearful faces presentation, and pleasant, unpleasant, and neutral sounds. The proposed paradigm for ex-COVID-19 patients should include cognitive tasks, such as mathematical tasks, n-back task, or Stroop task. In addition to the standard tasks for the study of memory processing and decision making, such paradigms should consist of images, sounds, and videos related to COVID-19 traumatic experiences. These paradigms would likely show differences in PFC activation on different tasks and might predict possible future mental disorders in vulnerable ex-COVID-19 patients.

### Facial and Oculometric Features

Distortions in facial and oculometric system have been reported in patients with various psychiatric disorders when compared to controls. These detected oculometric distortions can be categorized either as behavioral or as physiological depending on the extracted features. This section presents an overview of a limited number of studies that discuss prediction of psychiatric disorders from facial and/or oculometric features. This method is still in the discovery phase, but it has great potential, particularly due to non-invasiveness and inexpensiveness.

Behavioral changes include dysfunctions in oculomotor performance related to attention and fixation. Several studies have shown that attention deficit is a feature of psychiatric disorders such as depression, anxiety disorder, schizophrenia, and bipolar disorder (Bittencourt et al., 2013; Kleineidam et al., 2019). One often reported cognitive parameter for attention evaluation is saccadic eye movement. Patients with depression experienced shorter scanpath length in the free-viewing test, shorter duration of saccades and lower peak saccade velocity in the smooth pursuit test (Takahashi et al., 2021), as well as showed a greater latency, a reduction in movement precision, and a reduced peak velocity in the antisaccade task (Bittencourt et al., 2013; Li et al., 2016). Furthermore, individuals affected by PTSD and/or depression maintained their attention longer on the fearful, disgusted, and depressed expressions relative to the happy expression in comparison to controls (Sears et al., 2011; Armstrong et al., 2013; Lee and Lee, 2014; Powers et al., 2019). Additionally, PTSD patients demonstrated a greater number of initial eye fixations on the threat word and more orienting responses on all threatening and neutral words than controls (Felmingham et al., 2011; Powers et al., 2019).

Further, attention bias toward positive emotional faces studied in the laboratory has been shown to predict self-reported stress resilience (Thoern et al., 2016). We have developed and optimized general stimulation paradigms for elicitation of facial and eye gaze features for stress resilience assessment/prediction (Ćosić et al., 2016, 2019b).

Stress-induced physiological changes include dysfunctions in oculomotor performance exhibited in physiological parameters such as pupil diameter and spontaneous eye blink, which are modulated by ANS. Pupil diameter, modulated by the noradrenergic locus coeruleus (Rajkowski et al., 1993.), is reported to be larger in patients with PTSD exposed to threat-relevant stimuli and negatively valanced pictures (Kimble et al., 2010; Cascardi et al., 2015; Mckinnon et al., 2020). Previous studies have also indicated a relationship between spontaneous blink rate, a proxy of dopaminergic activity (Eckstein et al., 2017), and anxiety and/or depression (Mackintosh et al., 1983; Kojima et al., 2002; Al-gawwam and Benaissa, 2018).

Facial features that have been reported to be informative in classification of psychiatric disorders, especially depression, are facial expressions and face movement measures (Schultebraucks et al., 2020b; Stolicyn et al., 2020). Facial expressions used for prediction are in the literature described and supported by individual components of muscle movement, called Action Units (AUs) (Yao et al., 2021). There are 23 AUs all together, but some have stood out more than others in predicting psychopathological states (Gavrilescu and Vizireanu, 2019; Kumar et al., 2020). Beside facial expressions, face movement measures are also indicators of various disorders. A study by Jaiswal et al. (2019) included head pose in prediction of depression and anxiety, along with AUs and eye gaze but the discriminative power of the feature itself was not discussed.

The abovementioned studies did not include ex-COVID-19 patients, but the presented findings are useful in the context of prediction of psychopathological states in ex-COVID-19 patients, who reported increased symptoms of anxiety and depression and high risk of developing PTSD, sleep abnormalities, and cognitive impairments (Mazza et al., 2020; Wu C. et al., 2020).

The proposed protocol, based on the overview of the literature, should include clinical interview which would be video recorded and from which facial features then would be extracted. In addition to the recorded interview, the proposed protocol should also consist of oculometric paradigms such as antisaccade, free-viewing and smooth pursuit task, as well as images related to COVID-19 traumatic experiences, while the patients’ oculometric responses are being continuously recorded.

### The Proposed Prediction Protocol

**FIGURE 1:**
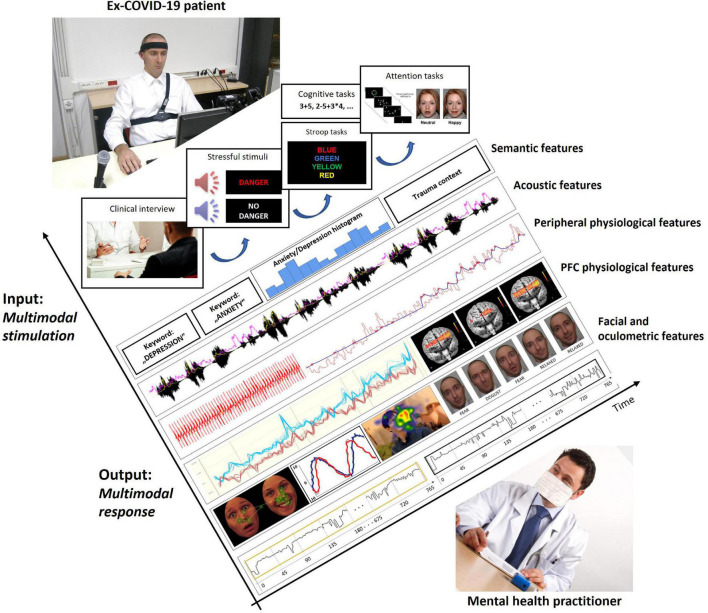
Illustration of time-synchronized multimodal input stimulation and multimodal output response of ex-COVID-19 patient during prediction protocol.

Based on the literature review and discussion of potential multimodal predictors of psychopathology in ex-COVID-19 patients, we first summarize the proposal of an experimental protocol for elicitation, acquisition, and assessment of the described multimodal data (Figure 1). The patients should first fill out a series of above listed self-report instruments. A relatively short structured clinical interview is suggested right after the self-report assessment. These two parts of the protocol can quickly and simply indicate the need of immediate psychiatric intervention in patients which are in the phase of emerging and evident psychopathology at the time of the screening. Furthermore, the clinical interview should be both audio and video recorded, similarly to a protocol recently used in deep-learning-based diagnosis of PTSD and depression following trauma (Schultebraucks et al., 2020b). The audio recording would therefore provide data for the extraction of various semantic and acoustic speech features, and the video recording would provide data for extraction of various video-based, i.e., facial/oculometric features. Patients which are classified as mentally healthy based on the self-report instruments and the clinical interview are administered the remainder of the experimental protocol, which should include: (a) a short and generic stimulation protocol for elicitation and assessment of physiological features of stress resilience (Ćosić et al., 2019a; Šarlija et al., 2021); and (b) a set of appropriate tasks for assessment of various fNIRS-based and attention-related features.

### Multimodal Data Fusion: Statistical and ML Analysis

The potential of AI in alleviating the consequences of the COVID-19 pandemic on mental health has already been recognized (Ćosić et al., 2020a,b; Thenral and Annamalai, 2020). The comprehensive data obtained through the proposed screening protocol of ex-COVID-19 patients can be analyzed by AI-based tools of unsupervised ML methods (e.g., multivariate correlation analyses, clustering, and principal component analysis), as well as a range of supervised ML methods (e.g., artificial neural networks, support vector machines, deep-learning supervised approaches). Such technologies are already being used in forecasting COVID-19 cases (e.g., Lalmuanawma et al., 2020; Santosh, 2020), as well as in COVID-19-specific (e.g., Prout et al., 2020; Herbert et al., 2021) and more general mental health context related to prediction of stress- or trauma-related adverse mental health outcomes from different types of data. For example, traditional supervised learning methods have been applied on: (a) self-report psychological measures and variables collected in emergency room to predict PTSD and depression (Galatzer-Levy et al., 2014; Karstoft et al., 2015; Ziobrowski et al., 2021); (b) bio-physio-clinical-demographic features from EHRs augmented by acute stress self-report measures to predict PTSD course (Galatzer-Levy et al., 2017; Schultebraucks et al., 2020a); (c) clinical and demographic features from EHRs to identify veterans at high suicide risk (Kessler et al., 2017); and (d) polygenic scores to predict the trajectory of depression development (Schultebraucks et al., 2021). Unsupervised clustering methods and physiological startle-based measures have been used in the long-term prediction of adverse psychological outcomes among physicians treating COVID-19 or non-COVID-19 patients (Dolev et al., 2021). Deep learning methods applied on facial and speech data have been shown to successfully classify PTSD and depression following a trauma (Schultebraucks et al., 2020b). The mentioned examples, as well as a recent review of 33 prognostic and 16 diagnostic studies on application of ML in trauma-related mental health disorders (Ramos-Lima et al., 2020), highlight the ability of ML techniques to improve not only diagnosis, but also to enable prediction of negative trauma-related mental health outcomes, which is important in the context of the current work.

**FIGURE 2:**
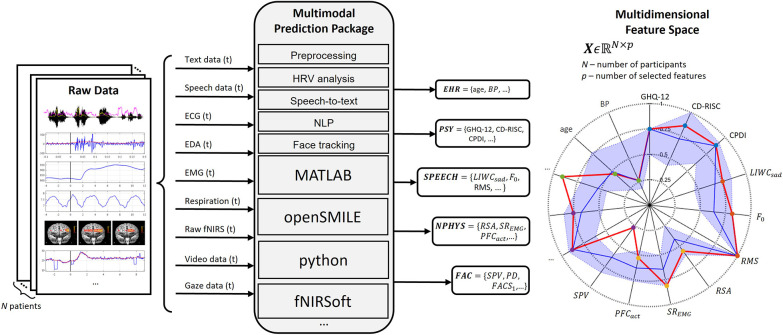
Multimodal data acquisition as input for real-time and offline feature computation. Illustrated subset of selected features includes: General Health Questionnaire (GHQ-12), Connor-Davidson Resilience Scale (CD-RISC), COVID-19 Peritraumatic Distress Index (CPDI); NLP feature LIWC “sad,” voice fundamental frequency (F_0_), voice root mean square (RMS); respiratory sinus arrhythmia (RSA), EMG-based startle reactivity (SR_EMG_), prefrontal cortex activity (PFC_act_); saccadic peak velocity (SPV), pupil dilation (PD), a feature related to facial action coding system (FACS).

Besides establishing a comprehensive set of potentially predictive features (illustrated in Figure 2), how we label the data deserves particular attention. The hierarchical data acquisition protocol described in the previous section would also be accompanied by a complex hierarchical fuzzy labelling system, which includes, e.g., PTSD risk (“high,” “medium,” “low”), depression risk (“high,” “medium,” “low”), state anxiety (“high,” “medium,” “low”). This labelling process requires longitudinal prospective research years after the initial measurement, which is feasible within the framework of post-COVID-19 clinics. The hierarchical data acquisition protocol should additionally result with a hierarchical prediction and decision support system, where preventive actions can truly be taken in a timely manner.

Methods based on supervised ML, while maximizing classification/prediction accuracy, often sacrifice model explainability and rigorous statistical validation. Accordingly, the need to focus on explainability, transparency, as well as generalizability of the ML models applied in psychiatry, by means of external validation of the developed models, has been recognized (Shatte et al., 2019; Chandler et al., 2020; Ramos-Lima et al., 2020). For example, only 6 out of 28 studies reviewed in 2019 by Graham et al. included external validation. One generalizability-related challenge for AI in mental health is dealing with prediction models for rare disorders or events, e.g., suicides in particular, which can generate unacceptably high number of false positive predictions once deployed on a large scale in practice, even when exhibiting high accuracies, sensitivities and specificities (Belsher et al., 2019; Jiang et al., 2020; Machado et al., 2021). Explainability, as a key prerequisite for the clinical use of the developed ML methods (Tjoa and Guan, 2020), is important for reliable prediction, but also for personalized treatment and prevention of mental health deterioration in ex-COVID-19 patients. When mentioning explainability, people often refer to global explainability (Jha et al., 2021), which is useful for understanding the underlying mechanisms of mental health deterioration, e.g., what variables contribute most significantly to a given prediction. Besides such global explanations, in the context of health applications, the local explainability of the developed models is crucial, i.e., providing the information to the end user, in this case the mental health practitioners, on why a particular decision was made. Examples of local explainability methods are LIME (Ribeiro et al., 2016) and SHAP (Lundberg and Lee, 2017). Such explanations for risk assessments and predictions are needed for mental health practitioners to justify their reliability, as well as to better tailor the preventive action for a given patient (discussed in the next section), according to the main determinants of the predicted negative mental health outcome. A more extensive discussion on the potential of explainable AI (XAI) in addressing the complexity of mental health research, particularly related to human-computer interaction, is available in Roessner et al. (2021). Furthermore, these new AI methods in prediction and prevention of mental health disorders should take into account human-computer interaction design principles on ethical, fair, trustworthy, and human-centered AI, which is important in the context of mental healthcare/suicide prevention and beyond (Xu, 2019; Robert et al., 2020). Additionally, other challenges like ethics due to sensitive data assessment, social factors, suitable education and training of medical professionals, as well as lack of evidence for clinical and economic impact of using AI in clinical psychiatric practice, are discussed in the literature (Graham et al., 2019; Aung et al., 2021; Lee et al., 2021).

## Prevention

Timely and appropriate preventive interventions among high-risk ex-COVID-19 patients, before the onset of serious psychopathology, should be of paramount common interest (Billings et al., 2020; Duan and Zhu, 2020). A variety of digital technologies which may assist in this globally increasing problem, including AI-based tools, are being discussed in the literature (Bickman, 2020; Ćosić et al., 2020b; Maulik et al., 2020; Gargot et al., 2021). Along these lines, the proposal in this section is based on our prior research work on tools and methods for prevention and treatment of stress-related disorders (Popovic et al., 2006; Ćosić et al., 2007, 2010a,2013a; Popović et al., 2009). The proposed preventive approach facilitated by AI tools is based on our research on an adaptive system for multimodal stimulation and multimodal real-time neuro-psycho-physiological feedback loop in the context of cognitive-behavioral exposure therapy (Rothbaum and Schwartz, 2002) and stress inoculation training (Meichenbaum, 2017), and is closely related to the emotion regulation framework (Gross, 1998), emotion regulation therapy (Mennin, 2006; Renna et al., 2017, 2020), as well as cognitive restructuring (Clark, 2013). Real-time processing of an individual’s multimodal neuro-psycho-physiological features, e.g., those presented in the previous section, is a prerequisite for a closed-loop emotion regulation strategy in the context of exposure therapy and stress inoculation training (Hodges and Rothbaum, 1998; Ćosić et al., 2010a); a similar closed-loop approach for a specific phobia has been demonstrated by Bãlan et al. (2020). Closing the loop over a single feature is commonly found in bio/neurofeedback (Choi et al., 2011; Tan et al., 2011), but more complex multimodal feedback has been more recently investigated in the context of fatigue and anxiety (Chang et al., 2016; Apostolidis et al., 2021). A study by Chang et al. (2016) demonstrated multimodal biofeedback in AI-based driver fatigue detection and alerting system implemented on an embedded real-time digital signal processing hardware, using six HRV parameters extracted from ECG signal and classification of yawning and eye closure events from face recordings. A study by Apostolidis et al. (2021) presented an AI-based biofeedback system for anxiety awareness, using anxiety classification from galvanic skin response, heart rate, and skin temperature signals on a low-cost real-time processing board and displaying relaxing pictures in cases of high anxiety. Both studies introduced AI methods into the multimodal feedback loop, but still used only one or two modalities and a limited number of features. In the context of mental health disorder prevention, more modalities and features are required, which would then require an optimal feature selection, faster AI models, as well as a higher computational power with an emphasis on real-time performance. As modalities and input complexity increase, so do real-time processing device requirements (Adams and Marketing, 2002), including sufficient number of cores for parallel processing and intensive computing, adequate amount of memory, high-speed interfaces capable of moving large amounts of data, and cost efficiency.

**FIGURE 3:**
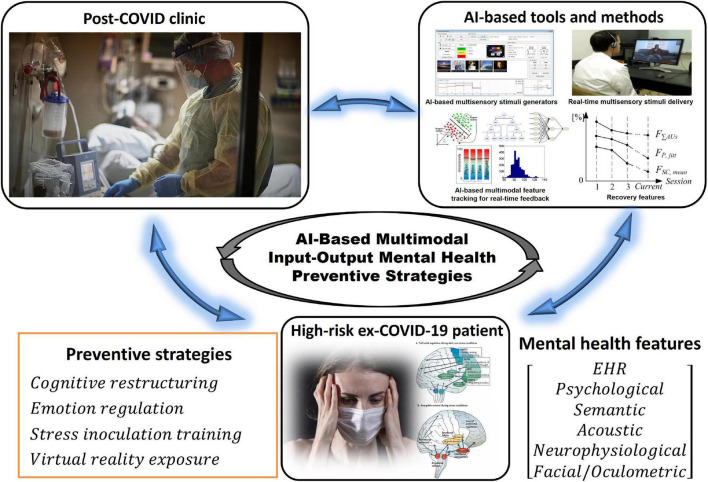
Concept of the integration of AI-based tools and methods with various preventive intervention strategies.

In this context, multisensory and multimodal interaction between the AI-assisted therapist and the treated ex-COVID-19 patient is based on a variety of customized multisensory inputs, such as non-interactive visual and auditory stimuli, interactive virtual reality stimuli and digital serious games (Li et al., 2014; Lau et al., 2017), as well as comprehensive multimodal neuro-psycho-physiological feedback features. Closed-loop control strategies, which match selected multisensory stimuli to multimodal feedback in real-time using a broad range of AI-based algorithms, from fuzzy logic (Popovic et al., 2006; Ćosić et al., 2007) to machine learning (Ćosić et al., 2020b), are related to different emotion regulation, exposure, stress inoculation and cognitive restructuring methods (Popovic et al., 2006; Ćosić et al., 2007, 2010a, b, 2011, 2013a, b; Popović et al., 2009). Conceptual illustration of the integration of AI-based tools with interventions relying on various psychotherapeutic preventive strategies is presented in Figure 3.

Patient-personalized *real-time multisensory stimuli generation* uses semantically and emotionally annotated pictures, natural and artificial sounds, recorded speech, written text messages, video-clips and films or virtual reality synthetic environments, which reflects the underlying complexity of potential chronic psychopathology. Usage of existing and dedicated large databases of images and/or sounds, which are semantically (Deng et al., 2009) and emotionally annotated (Horvat, 2017) *via* AI-tools related to ontologies (Horvat et al., 2009, 2012, 2014) and automated semantic segmentation (Chen et al., 2018), should optimize the delivery of the most relevant semantic and emotional stimuli content to the ex-COVID-19 patient during the intervention course, which is adapted to the patient’s reactions in real time (Ćosić et al., 2010a). *Real-time computation of multimodal neurophysiological, speech and facial/oculometric features*, as well as *real-time computation of optimal control multimodal closed-loop feedback* stimuli is a particular software and hardware challenge. Efficient search of comprehensive stimuli databases and selection of semantically and emotionally optimal stimuli within therapeutic context and strategy also deserve particular attention.

Comprehensive patient monitoring during emotion regulation in a multisensory and multimodal closed-loop feedback paradigm, based on real-time tracking of multidimensional neuro-psycho-physiological features, better reflects the complexity of the problem of stress-induced psychopathologies than focusing exclusively on conscious thinking process or a dysregulation of a single physiological variable. It is expected that augmentation of the number and variety of features to be computed in real time for closed-loop regulation definitely exceeds the capacities of any mental health professional. The presented AI-based approach, if available in post-COVID clinics after its scientific validation, may significantly relieve the overburdened mental health system.

Future research on personalized AI-based multisensory stimulation and multimodal feedback regulation may lead to novel digital clinical tools for more comprehensive patient screening, monitoring and reducing therapist burden, which is in line with calls for development and integration of new technology-enabled models of care to gain substantial improvements in patients’ mental health (Iorfino et al., 2021). An AI-assisted closed-loop psychotherapeutic approach within COVID-19 pandemic may also assist in lower cost, better and faster stratification of ex-COVID-19 patients according to high-medium-low risk of chronic psychopathology. Stratification of risk using the AI-based prediction methods, supplemented with personalized face-to-face risk assessments as necessary, can facilitate matching of ex-COVID-19 individuals with the most appropriate preventive psychotherapeutic intervention in severely resource-limited psychotherapeutic practices. For example, high-risk individuals might require the most effective first-line interventions with extensive involvement of the psychotherapist, while persons with moderately elevated risk might undergo validated online preventive intervention programs with minimal or no psychotherapist involvement. Prediction of risk will benefit from AI tools, as proposed in this paper, keeping in mind the necessity of future work on trustworthiness and generalizability of the developed methods, as discussed in subsection 2.6. On the other hand, the preventive intervention prescribed to a particular ex-COVID-19 patient based on his/her stratified risk might or might not use AI, depending on the selected intervention from the pool of validated preventive approaches that are available in a particular region. The proposed AI-based predictive and preventive approach should be viewed as a part of the whole package of measures focusing on protection of mental health during COVID-19 pandemic, which also include wide-ranging efforts emphasizing the protective potential of, e.g., physical exercise, sleep, and finding the sense of meaning in life (De Jong et al., 2020; Zhang Y. et al., 2020; Rajkumar, 2021), in times of physical distancing, isolations and other negative psycho-socio-economic consequences of attempts to limit the spread and mortality of the virus.

## Conclusion

The paper conceptually contributes to the accelerating state-of-the-art research in AI-based prediction of mental health disorders, particularly in ex-COVID-19 patients, calling for further coordinated large scale research efforts in prediction and prevention of mental health disorders, while integrating different categories of multidisciplinary data that have been shown promising in a growing number of separate research studies. The proposed AI-based methodology in prediction and prevention of negative psychological and behavioral consequences in ex-COVID-19 patients, potentially applied in newly established post-COVID clinics, deserves more attention. It expands traditionally used self-rating scales and psychiatric assessment questionnaires and interviews in diagnosis of mental health disorders with more objective multimodal neuro-psycho-physiological metrics based on state-of-the-art AI tools. The proposed prediction protocol may contribute to selection of post-COVID-stress most vulnerable individuals in immediate need for psychiatric treatment and psychological interventions. This approach might also be applied generally for all individuals who were exposed to higher levels of mental health risks during the COVID-19 pandemic and might assist in diagnostic process and in selection of the most appropriate preventive treatment strategies. Early prediction and effective treatment of ex-COVID-19 patients with the proposed methodology, particularly for most vulnerable individuals, is crucially important in prevention of widespread serious mental health diseases. The complexity of such interdisciplinary and multidisciplinary research deserves more global attention, particularly within the World Health Organization. Establishing a multinational interdisciplinary task force for policy design, planning and development of more advanced AI-based innovation in digital psychiatry may result in more successful and efficient strategies in coping with post-COVID overall mental health deterioration, leading to better, faster and more affordable global mental health services for all.

## Ethics Statement

Written informed consent was obtained from the individual(s) for the publication of any potentially identifiable images or data included in this article.

## Author Contributions

KĆ conceptualized the idea and design of the research study and wrote the first draft of the manuscript. SP, MŠ, IK, MG, BD, and IM wrote sections of the manuscript. TJ and NH provided critical revision of the work. All authors contributed to manuscript revision, read, and approved the submitted version.

## Conflict of Interest

The authors declare that the research was conducted in the absence of any commercial or financial relationships that could be construed as a potential conflict of interest.

## Publisher’s Note

All claims expressed in this article are solely those of the authors and do not necessarily represent those of their affiliated organizations, or those of the publisher, the editors and the reviewers. Any product that may be evaluated in this article, or claim that may be made by its manufacturer, is not guaranteed or endorsed by the publisher.
